# Ovarian Cancer Stroma: Pathophysiology and the Roles in Cancer Development

**DOI:** 10.3390/cancers4030701

**Published:** 2012-07-18

**Authors:** Mitsuko Furuya

**Affiliations:** Department of Pathology, Yokohama City University Graduate School of Medicine, Yokohama 236-0004, Japan; E-Mail: mfuruya@yokohama-cu.ac.jp; Tel.: +81-45-787-2587; Fax: +81-45-786-0191

**Keywords:** ovarian cancer, cancer stroma, chemokine, proinflammatory microenvironment

## Abstract

Ovarian cancer represents one of the cancers with the worst prognostic in adult women. More than half of the patients who present with clinical signs such as abdominal bloating and a feeling of fullness already show advanced stages. The majority of ovarian cancers grow as cystic masses, and cancer cells easily spread into the pelvic cavity once the cysts rupture or leak. When the ovarian cancer cells disseminate into the peritoneal cavity, metastatic nests may grow in the cul-de-sac, and in more advanced stages, the peritoneal surfaces of the upper abdomen become the next largest soil for cancer progression. Ascites is also produced frequently in ovarian cancers, which facilitates distant metastasis. Clinicopathologic, epidemiologic and molecular studies on ovarian cancers have improved our understanding and therapeutic approaches, but still further efforts are required to reduce the risks in the patients who are predisposed to this lethal disease and the mortality of the patients in advanced stages. Among various molecules involved in ovarian carcinogenesis, special genes such as *TP53*, *BRCA1* and *BRCA2* have been well investigated. These genes are widely accepted as the predisposing factors that trigger malignant transformation of the epithelial cells of the ovary. In addition, adnexal inflammatory conditions such as chronic salpingitis and ovarian endometriosis have been great research interests in the context of carcinogenic background of ovarian cancers. In this review, I discuss the roles of stromal cells and inflammatory factors in the carcinogenesis and progression of ovarian cancers.

## 1. Introduction

The ovary is a special organ that organizes menstruation, hormonal balance, bone metabolism and fertilization. Periodically, the adult ovarian stroma demonstrates active tissue remodeling during and after ovulation. In the women of reproductive ages and perimenopausal period, several pathologic events cause unfavorable conditions such as metrorrhagia, sterility and symptoms associated with climacteric disorders. Ectopic endometrial tissues in the ovary bleed periodically, causing longstanding inflammation, *i.e*., ovarian endometriosis. A series of investigations on ovarian neoplasms have improved our understanding of proinflammatory microenvironment including unfavorable cytokines, chemokines and imbalanced hormone production. Compared with the tumors of other organs, ovarian neoplasms are composed of more heterogeneous histologic types; not only epithelial tumors but also sex-cord stromal tumors and germ cell tumors frequently develop in this organ. Epithelial cells are lined only in monolayer on the surface, and they are exposed to mechanical stress such as periodical ovulation. The majority of ovarian parenchyma is composed of dense fibrotic stroma and follicles of various stages. Under physiologic hormonal milieu, follicular development, ovulation and luteinization are finely controlled. Therefore, stromal cells of adult ovary actively contribute to the synthesis and remodeling of extracellular matrix (ECM) and blood vessels.

Ovarian cancer represents one of the most common malignant conditions in adult women [1,2]. The macroscopic morphology of ovarian cancer is in most cases multi-cystic, regardless of histologic subtypes. On the other hand, the pathogenesis of ovarian cancers differs in histologic types, genetic backgrounds and pre-cancerous inflammatory conditions. Once the cysts rupture/leak and cancer cells are spread directly into the peritoneal cavity, disseminated cancer cells easily cause lethal peritonitis that involves the omentum and peritoneum, that is, peritonitis carcinomatosa. In this review, I introduce current understanding of ovarian cancers: (I) General view of adult human ovaries, (II) Pathologic backgrounds of ovarian cancers of four common histologic types, and in the latter sections, I highlight (III) Cancer stroma including ovarian parenchyma and peritoneal mesenchymal tissue. Cancer-related proinflammatory microenvironment is discussed from the points of cancer progression, angiogenesis and ascites production.

## 2. Constituent Cells of the Normal Adult Ovary

**Figure 1:**
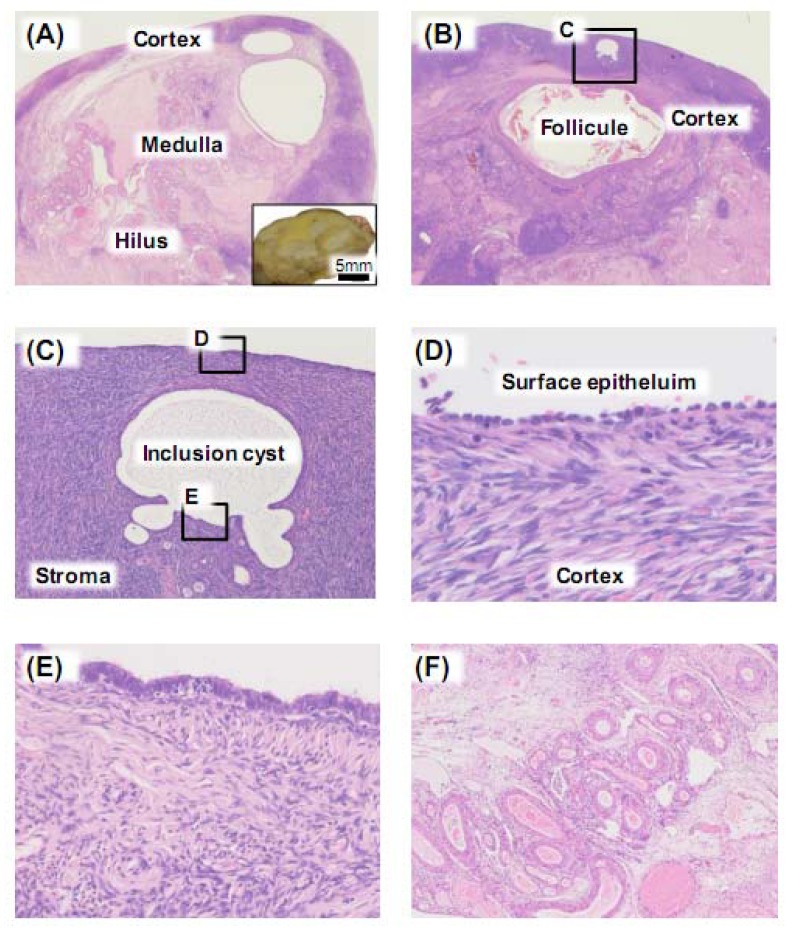
Macroscopic and microscopic features of the ovary. (**A**) Cut surface of the ovary. Cortex, medulla and hilus are distinguishable; (**B**) Intermediate magnification of cortex area; Rectangle (**C**) contains an inclusion cyst; (**C**) High magnification of cortex area indicated by rectangle in (**B**); Inclusion cysts are surrounded by dense fibrotic stroma; (**D**) High magnification of ovarian surface. Thin columnar surface epithelial cells are lined; (**E**) High magnification of an inclusion cyst. Tall columnar epithelial cells are lined; (**F**) High magnification of hilus area. Numerous vessels are accumulated.

From prepubertal to postmenopausal periods, the ovary exhibits a wide spectrum of appearance and functional activities. The adult ovary is composed of three zones: outer cortex, inner medulla and the hilus (Figure 1A). Follicular structures such as follicle and corpus luteum are located in the cortex (Figure 1B). After ovulation, the corpus luteum is gradually converted to a scar, *i.e*., the corpus albicans. Aged corpus albicans is either replaced by stroma or embedded in dense collagen fibers in the medulla.

The surface of the ovary consists of a single layer of peritoneal cells (Figure 1C,D). These surface cells are fragile and often invisible in resected ovaries. These mesodermally derived lining cells are not pure “epithelial” cells. They are immunoreactive for epithelial markers such as cytokeratin, Ber-EP4 and Wilms tumor gene (WT1) [3]. In addition, they are positively stained for mesothelial markers such as calretinin and vimentin. Therefore, these cells may be called “modified mesothelium/peritoneal cells”. Epithelial inclusion cysts are often observed in normal ovaries, and they are embedded in the cortex (Figure 1C), and infrequently, in the medullary stroma. The lining cells of inclusion cysts (Figure 1E) sometimes connect with ovarian surface cells, and are also stained for WT1 and other epithelial and mesothelial markers, supporting the notion that they are originated from surface lining cells. It is accepted that ovarian surface cells can undergo epithelial-mesenchymal transition (EMT) both *in vitro* and *in vivo*, especially in the process of post-ovulatory remodeling [4]. Although most of inclusion cysts remain inactive, the notion is widely accepted that inclusion cyst is potential precursor lesion of ovarian cancer. Biological dynamics of the cyst is great research interest among gynecologic oncologists. Several studies demonstrate differential molecular expression patterns between inclusion cyst-lining cells and quiescent surface cells [5,6,7].

Dysregulation of some transcription factors are noted in the formation of inclusion cysts, such that paired box gene 8 (PAX8) and CCAT/enhancer binding protein-β are upregulated, whereas GATA4 is frequently lost in inclusion cyst-lining cells [5,6]. Loss of the stroma-specific gene *Hoxa5* may also be involved. *Hoxa5* gene knockout mice tend to develop inclusion cysts in which cyst lining cells are strongly stained for PAX8 [7]. The finding indicates that Hoxa5 in ovarian stroma may play an important role in the physiologic regulation of ovarian surface epithelial cells.

Ovarian stroma is composed of spindle-shaped cells arranged in whorls or storiform pattern (Figure 1C). Other cells than these spindle cells include luteinized stromal cells. These luteinized cells may exist in the periphery of follicles and lutein cysts. They contain lipid, and look polygonal-shaped. These active stromal cells are generally indistinguishable from non-reactive stromal cells in shape, but they may produce steroid converting enzymes. Postmenopausal ovaries exhibit varying degrees of stromal cells, and it is often the case that hormonally inactive ovaries show relative stromal hyperplasia composed of fibroma-like spindle cells. Both the ovarian artery and the branch of the uterine artery penetrate ovarian hilus and supply the ovary with blood. Thus the ovarian hilus contains numerous blood vessels (Figure 1F). Apart from common epithelial tumors of the ovary, sex cord-stromal tumors originate from granulosa cells, theca cells, Sertoli cells, Leydig cells and fibroblasts of stromal origin. Hormonal disturbance is often observed in the patients with sex cord-stromal tumors because normal counterparts of these cell types produce estrogen, androgen and related steroids, which will be discussed later.

## 3. Pathogenesis of Ovarian Cancers

**Figure 2:**
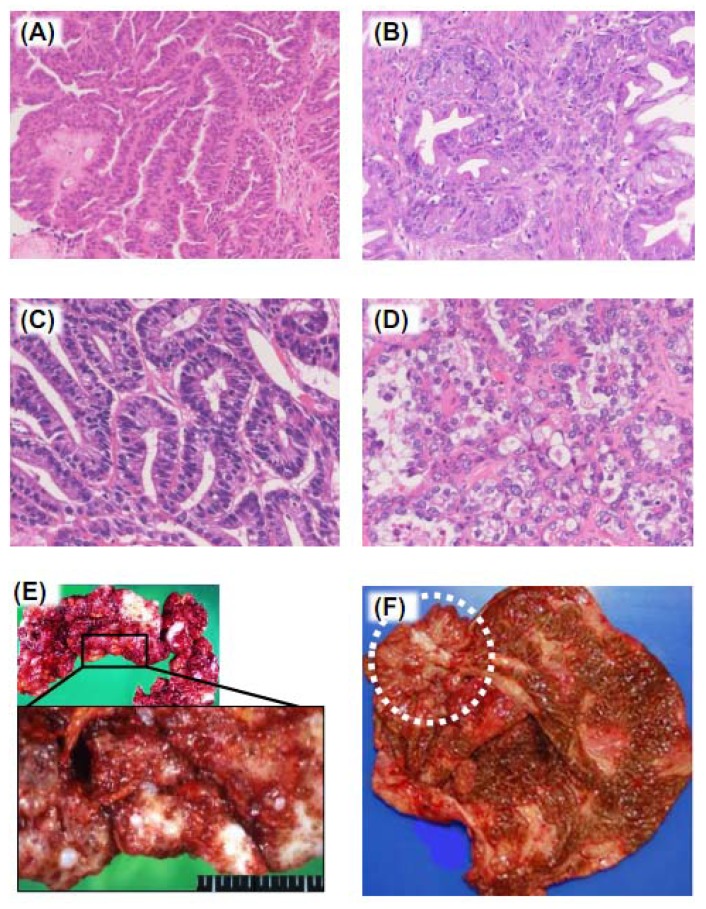
Histologic types of ovarian cancers. (**A**) Serous adenocarcinoma. The tumor shows papillary structure, and the cells do not contain intracytoplasmic mucin; (**B**) Mucinous adenocarcinoma. Irregular glands are composed of cell with atypical nuclei and intracytoplasmic mucin; (**C**) Endometrioid adenocarcinoma. The histology closely resembles endometrioid adenocarcinoma of the uterine corpus; (**D**) Clear cell adenocarcinoma. The cells have clear or eosinohpilic cytoplasm, protruding into glandular lumen; (**E**) Macroscopic feature of peritonitis carcinomatosa. The omental fat is completely replaced by metastatic cancer; (**F**) Macroscopic feature of EAOC in a chocolate cyst. Circle indicates cancer lesion.

Ovarian cancers belong to surface epithelial-stromal tumors that are the most common types among ovarian neoplasms. The etiology of sporadic ovarian cancers is not fully understood, but a series of investigations suggest that certain conditions such as genetic predisposition and benign inflammatory diseases are involved in the molecular mechanism of carcinogenesis. The pathologic studies have revealed differential background of ovarian cancers depending on histologic types [8,9]. There are four representative histologic types; serous, mucinous, endometrioid and clear cell types (Figure 2A–D). The other types such as transitional cell and squamous cell cancers are reported, if any, at low frequency. The ovarian surface is exposed to the peritoneal cavity. In most cases, cancer cells proliferate inward, and when thin cyst walls rupture, cancer cells spread into peritoneal cavity. Cancer cells may grow outward in papillary form, then directly expose themselves to the peritoneal cavity. Therefore, ovarian cancers preferentially metastasize to multiple surface sites of intraperitoneal organs including the omentum (Figure 2E). Hematogenous and lymphatic metastases are also associated with distant metastasis of this malignancy.

Serous cancer is divided into low- and high-grade subtypes [10,11]. Currently, it is widely accepted that low-grade and high-grade serous cancers develop through different carcinogenic pathways, respectively [9]. The former is characterized by mutations of *KRAS*, *BRAF *and *ERBB2* [11,12]. The latter is characterized by *TP53* mutations and not accompanied by mutations of *KRAS*, *BRAF* and *ERBB2 *[11,12,13]*.* Intensive studies on the pathogenesis of serous cancers have proposed that a majority of high-grade subtype may arise from the fimbriated end of the fallopian tube [14,15,16,17]. Ovarian surface is in close apposition with fimbria. Therefore, it may not be surprising that tubal intraepithelial cancer cells contact with ovarian surface and then subsequently destruct and replace the ovarian parenchyma, leading to “primary ovarian cancer” as a cystic mass. With regard to familial ovarian cancers, the histology of the patients with germline mutations in either *BRCA1* or *BRCA2* is in most cases serous type [18,19]. This may be consistent with the findings that serous type is the highest in frequency of tubal cancers, and that tubal cancers are found in a high number of patients with *BRCA *mutations who undergo prophylactic salpingo-oophorectomy [20].

Mucinous cancer is another frequent type of surface epithelial-stromal tumors. It requires careful clinicopathologic examination whether the ovary is either primary or metastatic. Advanced mucinous tumors spread and produce mucin lakes in the peritoneal cavity, *i.e*., pseudomyxoma peritonei. It is now widely accepted that the most cases of pseudomyxoma peritonei result from ruptured mucinous appendiceal adenomas/adenocarcinomas, and that the ovary is involved as the secondary lesion [8]. Metastatic ovarian cancers from the gastrointestinal tract frequently show mucinous architecture [21], thus it is critically important to define correctly the origin of the tumor by investigating the immunostaining patterns of cytokeratin 7, cytokeratin 20, CDX2 and some other diagnostic antibodies [22,23,24,25]. With regard to primary ovarian mucinous cancers, mutations of *KRAS *are observed at higher rates (46–75%) compared with the other types [26,27,28].

Endometrioid cancer may also show genetic changes in *PTEN* (20–43%) [29,30]. In addition, mutations of β-catenin are frequently observed in this type of tumor (32–64%) [29,31,32]. On the other hand, it is less well investigated the responsible genes contributing to the carcinogenesis of clear cell cancer. A recent study has revealed frequent mutations of chromatin remodeling gene *ARID1A* (*BAF250a*) in clear cell (57%) and endometrioid (30%) types of ovarian cancers [33,34]. Endometriosis, a chronic inflammatory disease, may exist in the vicinity of tumor nests of endometrioid and clear cell ovarian cancers in histology. Although the frequency of endometriosis lesion in these types of cancers varies among the studies, it is widely accepted that some endometrioid and clear cell ovarian cancers consecutively develop from the epithelial cells of endometriotic cysts (Figure 2F), which will be discussed later.

**Figure 3:**
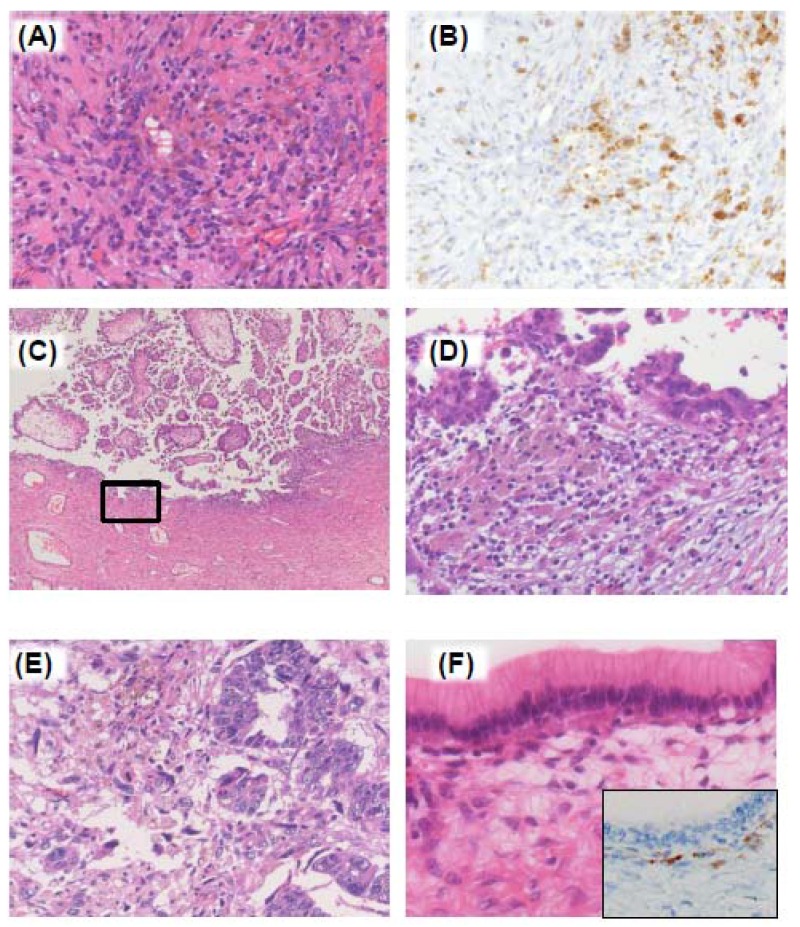
Cancer stroma. (**A**) Endometriosis. The ovarian stroma is infiltrated by numerous hemosiderine-laden macrophages, lymphocytes and plasma cells; (**B**) CD68 immunostaining is shown. Macrophages are highlighted; (**C**) Transitional lesion between endometriosis and EAOC; (**D**) High magnification of the rectangle indicated in (**E**). Lining epithelial cells show cytologic atypia; (**E**) Carcinosarcoma. Stromal component surrounding cancer nests demonstrates highly atypical nuclei, irregular morphology and often multi-nucleated appearance; (**F**) Functioning stroma. Stroma cells with clear cytoplasm are noted in the vicinity of epithelial glands. Inset: Functioning stroma cells are immunostained for Inhibin-α.

Apart from these ovarian cancers belonging to surface epithelial-stromal tumors, many other types of neoplasms develop in the ovary. Teratoma, dysgerminoma and yolk sac tumor are classified into germ cell tumors, and mature cystic teratoma (dermoid cyst) is the most common type of germ cell neoplasm of the ovary [35]. Stromal tumors, if not as common as surface epithelial-stromal tumors, account for 8% of ovarian neoplasms in the U.S. [36], and they exhibit characteristic features. The tumor cells of this group such as granulosa cells and theca cells may produce estrogen, androgen and related hormones [35]. Non-neoplastic counterparts of these cells are sometimes activated in the vicinity of cancer/adenoma glands, and they are called “ovarian tumor with functioning stroma” (Figure 3A), which will be discussed later.

The etiology of sex cord-stromal tumors and germ cell tumors is poorly understood. The mutation in *FOXL2* gene, which encodes a forkhead transcription factor, is reported to be present in granulosa cell tumors and less frequently in theca cell tumors with lutenization [37,38]. Germ cell tumors frequently occur to children and adolescent females, therefore, primitive germ cells that colonize the ovary during embryonic stage may aberrantly develop in the early decades of life.

## 4. Endometriosis-Associated Ovarian Cancer (EAOC)

As mentioned above, endometrioid and clear cell ovarian cancers occasionally co-exist with endometriosis (Figure 2F) [39]. Endometriosis is a major gynecologic disease that causes longstanding inflammation, pain and infertility. This disorder is pathologically defined as endometrial glands and stroma outside the uterus. Endometriosis primarily involves pelvic organs such as the peritoneum and ovaries, and the ovarian endometriosis may grow as the cysts containing menstrual effluent (chocolate cyst). The etiology of endometriosis remains to be the question under debate. The implantation theory (Sampson’s theory), that is, endometriosis is formed by implantation via retrograde menstruation, is widely accepted among physicians [40]. However, endometriosis of the other lesions than the urogenital system such as endometriosis in the lung and diaphragm cannot be explained solely by this mechanism. The metaplasia theory that proposes coelomic tissue transformation into endometrium [41], and the embryonic rest theory that explains Mullerian remnant differentiation into endometriotic cells, are also supported by some, if not all, gynecologic and pathologic research groups [42]. Although the pathogenesis may be different among the cases, clinicopathologic features are same; various types of immune cells including hemosiderin-laden macrophages infiltrate in endometriosis lesions where bleeding and tissue remodeling are repeated (Figure 3A,B). Inflammatory cytokines/chemokines are important in the progression of this disease. Ectopic endometrium demonstrates increased expression of various soluble factors, including heat shock protein 27, interleukin (IL)-1β, IL-6, IL-11, IL-12 and vascular endothelial growth factor (VEGF) [43,44,45]. Dysregulated immune responses are also noted. Natural killer (NK) cells show impaired function in the women with endometriosis [46,47,48]. T cells of both inflammatory and immunosuppressive subtypes are increased in the lesions and peritoneal fluid [45,49]. Although the profiles and functions of immune cells are not fully understood, a series of studies elucidate impaired immune responses in the local lesions of endometriosis.

The pathogenesis of endometriosis-associated ovarian cancers (EAOC) is, therefore, likely to be supported by endometriosis-based dysregulated inflammation. Relative risks for ovarian cancers among endometriosis patients are varied among reports [50]. It has been noted in several clinical studies that endometrioid and clear cell cancers are the most frequent histologic types preceded by endometriosis [50,51]. Incidence of EAOC is not fully understood precisely. It is because in part that the cancer tissue destructs and remodels its surrounding stroma in which previous endometriosis lesion is localized. A cohort study reported that cancer risk is elevated significantly among Japanese patients who have ovarian endometriosis, with standardized incidence ratios (SIR) of 8.95 (95% confidence intervals = 4.12–15.3) [52]. Swedish women with early diagnosed and long-standing endometriosis have a higher risk of ovarian cancer with SIR of 2.01 and 2.23, respectively [53]. Not only clinical data but also histopathologic findings support the notion that endometriosis stands as a precursor lesion of ovarian cancer [54,55]. Atypical endometriosis, namely that atypical cells lining in endometriotic cyst and atypical endometrial hyperplasia arising in ovarian endometriosis, is occasionally observed in the vicinity of EAOC (Figure 3E,F). With regard to epithelial abnormalities, molecular studies have revealed that ovarian cancer cells and coexisting endometriosis epithelium share common methylation pattern of the androgen receptor gene and loss of heterozygosity (LOH) at tumor suppressor gene loci [56,57,58]. Since the lesions are exposed to special hormonal and inflammatory factors associated with endometriosis, the role of stroma in the carcinogenesis of EAOC of endometrioid and clear cell types should be distinguished from that in advanced serous cancers with *BRCA1 *and/or *TP53* mutations.

## 5. Roles of Stroma for the Development of Ovarian Cancers

Cancer tissues preferentially generate special stroma for aberrant proliferation and invasion. The fibroblasts and some other cell types such as pre-existing vascular cells and mesenchymal stem cells potentially become cancer-associated fibroblasts (CAF) [59]. Distinctive genetic abnormalities were reported in CAF of some cancer types [60,61,62]. On the other hand, some other studies found no aneuploidy [63,64]. The genetic alteration in CAF of ovarian cancers was reported, such as allelic imbalance on chromosome 3p21 and 18q21 [65,66]. A later study, however, concluded that somatic genetic alterations are extremely rare in CAF of ovarian cancers [67]. Qiu *et al*., in the latter study, suggested that the quality of DNA accessible from paraffin-embedded tissues may not be reliable for the assessment of genetic alterations [67].

Several cytokines activate fibroblasts, such as hepatocyte growth factor (HGF), fibroblast growth factor-2 (FGF-2), transforming grow factor (TGF)-β, CXCL12 and platelet-derived growth factor (PDGF) [59]. TGF-β produced by ovarian cancer cell line SKOVA3 activates omental fibroblasts [68,69], and direct interaction between the cancer cells and fibroblasts promotes invasion through increased expression of HGF and matrix metalloproteinase (MMP)-2 [68]. The stroma in ovarian cancers should be considered in the light of progression stages. The ovarian cancer cells in advanced stages can be a double-edged sword; they do not only infiltrate for local invasion but also disengage stromal interaction for dissemination. In this review, some important stromal components are highlighted.

### 5.1. Extracellular Matrix (ECM)

The dynamics whereby cancer cells detach from the primary site and attach to the secondary sites are closely associated with the cross communication between cancer cells and ECM such as collagen and laminin. Ovarian cancer cells may alter the expression of surface receptor for ECM and increase the mobility [70], and may also produce cancer-favorite ECM by themselves [71,72]. Several integrin complexes serve as the cell surface receptors for laminin. For example, α6β4-integrin provides the linkage between intracellular keratin filament to laminin. α3β1-integrin controls actin-based cytoskeletal rearrangement at cel-cell boundaries. Both upregulation and downregulation of integrin subunits were reported in advanced cancers, and the alterations may depend in part on gene expression levels in cancer cells [73,74,75,76]. In addition, some scaffold proteins such as tetraspanins CD9, CD82 and CD151 also mediate the function of integrins [77,78]. We demonstrated that CD9 and CD151 mediate cellular localization of α6β4-integrin and several β1-integrin subsets on some tumor cell lines including an ovarian cancer cell line HTOA [79,80]. The expression levels of these integrin subunits were not reduced significantly, but they withdrew from cell surface. Dynamic re-distribution of integrins and downregulation of MMPs were observed [80], indicating that integrin-mediated cell-ECM adhesion system and cell-cell boundaries are finely controlled by these tetraspanins, leading to migration and dissemination.

Differential expression profiles of MMPs and tissue inhibitors of MMPs (TIMPs) were reported according to ovarian cancer histology [81,82,83,84]. Proteolytic enzymes are produced not only cancer cells but also stromal components. We previously reported that desmoplastic stroma of ovarian cancers expressed MMP-2 and MMP-9, but not MMP-7 [81]. Although normal ovarian stroma such as lutein cells and mesothelial cells potentially produce these MMPs, the expression levels, the activation of MMPs, and the balances of TIMPs/MMPs may reflect histologic types and malignancies [85]. Histopathologic investigations of MMPs revealed that MMPs-associated proteolysis activities are detectable only in the stroma, not in cancer cell nests in some cases [81]. The results indicate that CAF may play dominant roles in invasion-related ECM remodeling in some cases, if not all, of ovarian neoplasm [81]. MMPs also accelerate angiogenic microenvironment of ovarian cancer by activating VEGF [86]. Most ovarian cancers grow as cystic masses, and cyst fluids produced by cancer cells contribute to active remodeling of ovarian ECM [85,87]. Among aforementioned histologic types, mucinous tumors produce a large amount of mucous substances and form mucin lakes in the stroma. Even if tumor cells are not present there, the mucin *per se* contains several types of MMPs that destruct pre-existing ovarian stroma [85].

### 5.2. Mesothelium

The peritoneal cavity is lined by mesothelial cells, and ovarian cancer cells spread, interact and are implanted in mesothelial cells. Therefore, the molecules that mediate cancer cell-mesothelium binding are of great interest as possible targets for new therapeutic approaches. The mesothelium expresses glycosylphosphatidylinositol (GPI)-anchored glycoprotein, namely mesothelin, on the cell surface [88]. Several studies elucidated the importance of mesothelin as a therapeutic target [89,90,91]. CA125 (also called MUC16) is the most commonly used biomarker in ovarian cancer. It has both membrane-bound and secreted forms, and the latter form is present abundantly in ovarian cancer patients’ circulation. Because CA125 is a very large molecule and heavily glycosylated, the biological properties of this glycoprotein had not been well understood previously. Intensive studies clarified the binding of ovarian cancer cells expressing CA125 to mesothelial cells via mesothelin [92,93]. Inhibition of this biding was shown to abolish the adhesion of ovarian cancer cells to mesothelin-expressing cells. Phase I clinical trial of mesothelin-targeted therapy has been done [94], and the improvement and validation of effective blocking of cancer cells binding to mesothelium are awaited.

### 5.3. Omental Adipose Tissue

Apart from the ovarian stroma that initially contacts with primary cancer cells, the stroma of metastatic lesions also play important roles in disease progression. The omentum is one of the most common metastases sites of ovarian cancers (Figure 2E). Omental adipocytes abundantly secrete IL-6, CXCL8, CCL2, TIMP-1 and adiponectin. Nieman *et al*. demonstrated that these factors facilitate migration of ovarian cancer cells to omental adipocytes [95]. Direct interaction between ovarian cancer cells and adipocytes results in the transfer of fatty acids to ovarian cancer cells and alters intracellular metabolic activities. Activated ovarian cancer cells by adipocytes highly expressed fatty acid-binding protein 4 (FABP4). FABP4-depleted mice showed significant reduction in tumor progression and metastases [95]. These results elucidate the role of omental adipocytes as special stroma for ovarian cancer dissemination.

### 5.4. Malignant Stroma and Functioning Stroma

Ovarian cancers, especially endometrioid type, are infrequently accompanied by biologically malignant stroma, so called “carcinosarcoma” (Figure 3E). Fujii *et al*. investigated LOH in the carcinosarcomas of the ovary and uterus [96]. They micro-dissected carcinoma and sarcoma lesions, respectively, and analyzed allelic status of several chromosome arms. They demonstrated that sarcomatous components arise secondarily from epithelial components by successive genetic changes, but not *vice versa* [96]. This finding may be interpreted as a distinctive pattern of EMT that leads to malignant transformation of mesenchymal components. In uterine neoplasms, LOH is a frequent event in endometrial stromal sarcomas [97]. Therefore, it may be the case that the stroma of ovarian cancers of endometrioid type potentially exhibits genetic alteration as they progress.

One of special properties of ovarian stroma interacting with cancer/pre-cancerous cells is functioning stroma that produces steroid hormones (Figure 3F). The functioning stroma contains lutenized mesenchymal cells. Although it is not concluded whether increased estrogenic or androgenic hormones accelerate ovarian cancer development, a few studies suggest that hormone therapy, especially estrogen alone, increases the risk of ovarian cancer [98,99]. Sex cord-stromal tumors occasionally induce endometrial hyperplasia and adenocarcinoma of the uterus. Ovarian cancer cells of endometrioid type frequently express estrogen receptor. Therefore, cancer cells of this type may take advantage of functioning stroma-derived estrogen for development.

## 6. Key Signaling Molecules Involved in Proinflammatory Microenvironment of Ovarian Cancers

The inflammatory microenvironment of ovarian cancers varies among cases, depending on disease background, clinical stages, cancer immunogenicity, host immune response, and repertoire of cytokines/chemokines produced by both tumor and stromal sides. Once cancer cells spread into the peritoneal cavity, they potentially invade peritoneum and omentum, causing carcinomatous peritonitis. Such systemic inflammation does not necessary mean anti-cancer reaction because cancers may cause their favorite inflammation by taking advantage of immunosuppressive cells including regulatory T cells (Treg) and tumor associated macrophages (TAM). Some studies demonstrated that Treg are recruited to ovarian cancer lesions, leading to poor prognostic outcomes [100,101].

In advanced ovarian cancer cases, uncontrollable amounts of ascites are occasionally produced. Several angiogenic and pro-tumoral cytokines/chemokines in ascites have been investigated as prognosis-prediction markers [102,103,104,105]. Chemokines are a family of low molecular weight cytokines, and they exert the activities by binding to corresponding G protein-coupled receptors (GPCRs). There are CC, CXC, C and CX3C subfamilies in human based on the spacing or the presence of four *N*-terminal cysteine residues, and most members are classified into either CXC or CC subfamilies [106,107]. I highlight CXC and CC chemokines and a few other molecules.

### 6.1. CXC Chemokines

CXC chemokines with Glu-Leu-Arg (ELR) motif (ELR^+^), such as CXCL1 (GRO-α), CXCL2 (GRO-β), CXCL3 (GRO-γ), CXCL5 (ENA-78), CXCL6 (GCP-2), CXCL7 (NAP-2) and CXCL8 (IL-8), work as potent angiogenic factors [108,109]. CXCR2 works as a primary functional receptor for these ELR^+^ chemokines [110]. A study showed that stroma-derived MMP1 activate protease-activated receptor-1 (PAR1) expressed in ovarian cancer cells, and the epithelial-stromal crosstalk induces the secretion of CXCL1 and CXCL8 from cancer cells [111]. These chemokines exhibit angiogenic properties, and accelerate disease progression [111]. Another study revealed that CXCL8 is inducible in cancer cells, and that it is highly produced and processed as *N*-terminally truncated form in the ascites of ovarian cancer patients [112]. IFN-γ-inducible CXC chemokines without ELR motif (ELR^−^), such as CXCL9 (Mig), CXCL10 (IP-10) and CXCL11 (I-TAC), are considered to be antiangiogenic factors [110]. In ovarian cancer, These ELR^−^ chemokines share a common receptor CXCR3 [113,114]. CXCR3-A is a fundamental receptor for CXCL9, CXCL10 and CXCL11. CXCR3-alt interacts only with CXCL11 whereas CXCR3-B works as a functional receptor for another ELR^−^ chemokine CXCL4 (PF-4). CXCL4 also interacts directly with VEGF and FGF-2, exerting inhibitory effects on these factors [115,116,117]. Although CXCR3 is expressed in cytotoxic T cells, several studies demonstrated that CXCL9 and CXCL10 induced migration of CXCR3^+^ tumor cells [118,119], suggesting that CXCR3^+^ tumor cells preferentially metastasize to lymph nodes expressing these ligands [118]. In ovarian cancers, we found that CXCL11-CXCR3-A/CXCR3-alt axes are increased and that CXCL4-CXCR3-B axis is decreased in ovarian cancers [120]. In the EAOC lesion developed in endometriosis cyst, the expression level of CXCL4 is significantly lower than that in corresponding endometriosis [121]. The findings indicate differential inflammatory milieu between endometriosis and the cancer developing on the bases of endometriosis.

Another ELR^−^ CXC chemokine CXCL12 binds to CXCR4. CXCL12-CXCR4 axis plays protumoral roles in many types of cancers, and intensive studies have been done to explore the therapeutic approaches that target CXCR4-mediated cancer invasion and metastasis. Ovarian cancer cells express CXCR4 [122] and consistently, high concentrations of CXCL12 are detectable in the ascites and tumor masses of ovarian cancer patients [123,124], suggesting that this axis accelerates cancer dissemination in the peritoneal cavity. CXCL12 crosstalks with the prime angiogenic molecule VEGF and synergistically induce strong angiogenesis [124]. It should be noted that CXCL12 expresses not only in advanced stage ovarian cancers but also in Stage I and II cancers [124].

### 6.2. CC Chemokines

CXC chemokines can exert direct effects on neutrophils and endothelial cells, whereas CC chemokines preferentially interact with monocytes that express corresponding receptors. Several CC chemokine members, including CCL2 (MCP-1), CCL3 (MIP-1α), CCL5 (RANTES), CCL7 (MCP-3), CCL8 (MCP-2), CCL17 (TARC) CCL 20 (MIP-3α), CCL22 (MDC) and CCL23 (MPIF-1), are produced mainly by tumor infiltrating lymphocytes (TIL) and monocytes, and some of them also by cancer cells. For example, CCL22 produced by ovarian cancer cells plays a key role in the recruitment of immunosuppressive Treg [100]. CC chemokines attract circulating mesenchymal cells and cancer cells expressing corresponding receptors, and the axes accelerate angiogenesis and metastasis. Milliken *et al*. investigated the profile of CC chemokines and their receptors in the ascites of ovarian cancers [102]. They found that CD3^+^CD4^+^ T cell populations and CD14^+^ macrophages in the ascites preferentially express CCR2 and CC5R, suggesting that they play important roles in CC chemokine networks of ovarian cancer ascites. Another study demonstrated that biologically active CCL2 is detected at significant level in the ascites of ovarian cancer [112]. Furthermore, the latter study showed that processed form of CCL18 produced by monocytic population including TAM is significantly elevated in the ascites [112]. On the other hand, a different group investigated CC chemokine-associated microenvironment in ovarian cancer stroma and ascites, and found defective CCR2 expression [125]. The impaired CCL2-CCR2 axis in their cases seems to be dependent on local TNF production [125]. CCL5 produced by CD4^+^ T cells activated CC5R^+^ dendritic cells, eliciting tumor-reactive CD8^+^ T cell to kill ovarian cancer cells through CD40. This therapeutic benefit was not obtained using CCL5^−/−^ T cells [126]. These inconsistent results should be further investigated from the viewpoint of histologic types and disease backgrounds of enrolled cases. As I discussed previously, some ovarian cancers develop on the bases of genetic predisposition, and some other cancers develop on the bases of chronic inflammations. In the latter condition, ascites may contain disease-dependent inflammatory factors, indicating that the properties of immune cells may be already skewed at the initial period of dissemination.

### 6.3. Other Molecules

Angiogenic molecules play critical roles in ovarian cancer progression and metastasis. Among numerous important factors that control vessel remodeling, VEGF and VEGF receptors (VEGFRs)-related pathways are widely investigated as landmark targets in clinical antiangiogenic trials. The elevated VEGF is associated with poorer survival in ovarian cancers [127,128]. Preoperative serum VEGF levels become an independent survival factor, and are associated with the presence of ascites [129]. These clinical studies suggest that VEGF not only in the local lesion but also in the ascites and serum contribute to cancer progression. VEGF exerts angiogenic cascades via its main receptor VEGFR-2. The downstream molecules include Ras/Raf/MAPK, PLC-γ that hydrolyzes PIP2 and activate PKC, and PI3K that activates Akt. Signaling pathways through Ras/Raf/MAPK and PKC generally induce increased permeability, proliferation and migration of endothelial cells, and those through PI3K control survival signals [130]. In addition to these direct effect, VEGF also crosstalks with some other important molecules such as MMPs [86] and CXCL12 [124], exerts synergistic angiogenic effects, as I mentioned before.

Many other molecules are involved in inflammatory milieu of ovarian cancers. I introduce some recent landmark studies among them. The net valance between Treg and IL-17-producing helper T cells (Th17) is critical in immune responses against cancer. Miyahara *et al*. investigated Th17-mediated cytokine network in ovarian cancers. They suggest that the relative concentration of TGF-β, IL-1 and IL-6 in cancer milieu may determine the fate of CD4^+^ T cells into Treg or Th17 [131]. Kryczek *et al*. investigated Th17 in more than 200 human ovarian cancer samples. They demonstrated that the levels of infiltrating Th17 cells and ascites IL-17 are reduced in advanced cancer cases, and that IL-1β stimulates Th17 cells for IL-17 production and recruitment of cytotoxic T cells [132]. Another study proposes that IL-2 may play a key factor that converts Treg into Th17 in ovarian cancers, enhancing antitumor immunity [133]. All these studies discuss IL-17 in the view that IL-17 contributes to anti-cancer inflammation, but it should be further investigated whether Th17-mediated proinflammatory signaling network actually exhibits anti-cancer property in both localized and advanced ovarian cancers of all histologic types. A number of studies demonstrated pros and cons of the presence of IL-17 in cancer progression [134,135,136,137], indicating that the role of IL-17 is context-dependent.

Buckanovich *et al*. isolated tumor associated vascular endothelial cells by microdissection from TIL-rich ovarian cancers and TIL-lacking ovarian cancers, respectively [138]. They found that *endothelin B receptor *(*ETBR*) gene is upregulated in the latter group [138]. ETBR belongs to GPCRs, and its blockade restores T cell adhesion to cultured endothelial cells *in vitro* and increases TIL homing to tumor lesion *in vivo*. The mechanism is explained in part that ETBR antagonist suppresses NO-release and upregulates ICAM-1 in endothelial cells, which increases TIL binding and infiltration [138]. This study may explain a possible mechanism how tumor vessels help tumor escape from immunotherapy.

Collectively, skewed immune cells such as TAM and Treg, special angiogenic vessels such as tumor-associated endothelial cells, and various proinflammatory cytokines/chemokines including CCL18 and CCL22 are synergistically involved in the suppression of anti-tumor immunity.

## 7. Conclusions

In this review, the pathophysiology of ovarian cancers was discussed from the points of stroma and proinflammatory milieu. As most advanced ovarian cancers spread in the peritoneal cavity and grow in metastatic sites, “stroma of advanced ovarian cancer” should be considered in a wide range of view, including mesothelium, adipose tissue and cancer-producing fluid. Organ-specific stroma with the potency of hormonal production, proinflammatory molecules and peritoneal mesenchyma to which cancer cells migrate are all important factors for better understanding of the dynamics of ovarian cancers.

Apart from common epithelial-stromal ovarian cancers, peritoneal cancers so called “normal-sized ovary cancer syndrome” exhibit similar course to ovarian and tubal serous counterparts [139]. Cancer cells of this type show serous papillary morphology, and are positively immunostained for Ber-EP4 and WT1, and indistinguishable from serous ovarian cancers by cytology and immunohistochemistry [140,141]. Molecular analyses revealed that peritoneal cancers originate in a multifocal manner [142]. Germline mutations of *BRCA1 *are also associated with this type of cancer at a high rate [143]. Other malignant and pre-cancerous conditions with ovarian histologic subtypes also occur to the peritoneum such as endometriosis, EAOC and mucinous adenocarcinoma [144,145].

Series of investigations have improved our understanding of the nature of ovarian cancers. Many important findings have unveiled distinctive differences in the pathogenesis and microenvironment depending on histologic types and pre-cancerous conditions. Meanwhile, the complexity of proinflammatory milieu has also been elucidated. Further studies are necessary on ovarian cancer stroma including vasculature, inflammatory cells and mesenchymal components of the peritoneal cavity in different types and stages, which will improve the prognosis of the patients by exploring the frontier of more effective and safer therapeutic strategies.
